# GQ262 Attenuates Pathological Cardiac Remodeling by Downregulating the Akt/mTOR Signaling Pathway

**DOI:** 10.3390/ijms251910297

**Published:** 2024-09-25

**Authors:** Haoyue Ma, Yang Ge, Chang Di, Xin Wang, Boyang Qin, Anhui Wang, Weipeng Hu, Zirui Lai, Xiaofeng Xiong, Rong Qi

**Affiliations:** 1 Department of Pharmacology, School of Basic Medical Sciences, Peking University Health Science Center, 38 Xueyuan Road, Haidian District, Beijing 100191, China; haoyue.ma888@gmail.com (H.M.);; 2 State Key Laboratory of Vascular Homeostasis and Remodeling, Peking University, Beijing 100191, China; 3 NHC Key Laboratory of Cardiovascular Molecular Biology and Regulatory Peptides, Peking University, Beijing 100191, China; 4 Beijing Key Laboratory of Molecular Pharmaceutics and New Drug Delivery Systems, Peking University, Beijing 100191, China; 5 State Key Laboratory of Natural and Biomimetic Drugs, Peking University, Beijing 100191, China; 6 State Key Laboratory of Anti-Infective Drug Discovery and Development, School of Pharmaceutical Sciences, Sun Yat-sen University, Guangzhou 510006, China

**Keywords:** GQ262, Gαq/11, cardiac hypertrophy, fibrosis, apoptosis, Akt/mTOR

## Abstract

Cardiac remodeling, a critical process that can lead to heart failure, is primarily characterized by cardiac hypertrophy. Studies have shown that transgenic mice with Gαq receptor blockade exhibit reduced hypertrophy under induced pressure overload. GQ262, a novel Gαq/11 inhibitor, has demonstrated good biocompatibility and specific inhibitory effects on Gαq/11 compared to other inhibitors. However, its role in cardiac remodeling remains unclear. This study aims to explore the anti-cardiac remodeling effects and mechanisms of GQ262 both in vitro and in vivo, providing data and theoretical support for its potential use in treating cardiac remodeling diseases. Cardiac hypertrophy was induced in mice via transverse aortic constriction (TAC) for 4 weeks and in H9C2 cells through phenylephrine (PE) induction, confirmed with WGA and H&E staining. We found that GQ262 improved cardiac function, inhibited the protein and mRNA expression of hypertrophy markers, and reduced the levels of apoptosis and fibrosis. Furthermore, GQ262 inhibited the Akt/mTOR signaling pathway activation induced by TAC or PE, with its therapeutic effects disappearing upon the addition of the Akt inhibitor ARQ092. These findings reveal that GQ262 inhibits cardiomyocyte hypertrophy and apoptosis through the Akt/mTOR signaling pathway, thereby reducing fibrosis levels and mitigating cardiac remodeling.

## 1. Introduction

With high morbidity and mortality, cardiovascular diseases have been one of the major public health concerns [1]. Cardiac remodeling usually refers to changes in genomic expression, molecular expression, cardiac cells and their interstitium, and is clinically characterized by corresponding changes in size, shape, and function after cardiac injury [2]. The cellular-level changes resulting from cardiac remodeling include cardiac hypertrophy, necrosis, apoptosis, and fibrosis [3]. The myocardium undergoes hypertrophy to cope with acute overload, initially described as a compensatory mechanism to reduce wall stress during acute overload [4]. Maladaptive cardiac hypertrophy leads to the development of heart failure (HF).

G proteins are typically considered molecular switches that mediate signal transduction by linking seven transmembrane G protein-coupled receptors (GPCRs) to various intracellular effectors [5]. G proteins serve as critical nodes on the classical GPCR/G protein/effector axis and participate in a wide range of human pathological and physiological processes [6]. GPCRs have been identified as therapeutic targets for cardiovascular diseases such as myocardial infarction (MI) and atherosclerosis (AS) [7,8]. One of the characteristics of cardiac hypertrophy is the amplification of Gαq/11 signaling [9,10]. Activation of various GPCRs, such as angiotensin II type 1 receptor (AT1R) and β2-adrenergic receptor (β2AR), can trigger Gαq signaling. The GPCR signaling pathways, including diacylglycerol (DAG) and protein kinase C (PKC), play a critical role in myocardial hypertrophy [11]. A slight overexpression of wild-type murine Gαq in the mouse heart resulted in cardiac hypertrophy. This condition was marked by a conserved fetal gene expression program, an elevated heart-to-body weight ratio, and enlarged cardiomyocytes [12]. Myocardial hypertrophy induced by Gαq notably compromised systolic cardiac function, and at elevated expression levels it led to dilated cardiomyopathy accompanied by pronounced cardiac failure [12]. After inhibiting the downstream activation of Gαq/11 by transgenic mice, the increase in heart weight following transverse aortic constriction (TAC) was abolished [13]. Phenylephrine (PE) is a commonly used inducer for inducing cardiomyocyte hypertrophy. PE primarily couples with the Gαq/11 subfamily of G proteins [14]. PE stimulation triggers the hydrolysis of phosphatidylinositol 4,5-bisphosphate, producing inositol 1,4,5-trisphosphate and DAG. DAG acts as a potent activator of PKC, and these changes can further aggravate the pathological progression of cardiac remodeling [15]. Although the critical role of Gαq/11 in cardiac hypertrophy has been established, research on Gαq in cardiovascular diseases remains limited compared to Gαs and Gαi [16,17].

Currently, there are only a few Gαq/11 inhibitors available, such as GQ127 and natural Gαq/11 inhibitors, FR900359 and YM-254890 [18,19]. Existing inhibitors have insufficient inhibitory effects on Gαq/11 and are limited by their low abundance in nature, synthetic complexity, toxicity and side effects caused by non-specificity. Consequently, no small-molecule Gαq/11 inhibitors are currently used for treating cardiac remodeling. These considerations have prompted us to explore novel small molecules with enhanced inhibitory potency on Gαq/11 and with favorable drug-like properties of good safety and augmented pharmacokinetics [20], just like GQ262, a novel Gαq/11 inhibitor developed by our collaborator Prof. Xiong’s team [21]. GQ262 was developed through the design and structural optimization of the previously synthesized inhibitor GQ127 [20]. The modifications included reducing the carbonyl group and cyclizing the head fragment to enhance potency and drug-like properties. Inositol monophosphate (IP1) assay results showed that GQ127 had an IP1 inhibition of 36.5 ± 0.3% at 10 μmol/L [20], whereas GQ262 achieved 57.2 ± 1.9% inhibition of Gαq/11, indicating that GQ262 has stronger inhibitory efficacy against Gαq/11. We are curious whether this Gαq/11 inhibitor can be effective in preventing cardiac hypertrophy.

Cardiomyocyte apoptosis plays a crucial role in the exacerbation of HF, and therefore significant pathophysiological implications for the onset and progression of the diseases [22]. Several studies have explored the molecular mechanisms of Gαq/11-induced apoptosis. It has been found that Gαq-induced apoptosis depends on PKC in COS-7 and CHO cells [23]. Additionally, angiotensin II (Ang II)-induced myocyte apoptosis relies on increased intracellular Ca^2+^, indicating the involvement of the Gαq/11-phospholipase C pathway [24].

The Akt/mTOR signaling pathway is one of the downstream pathways of Gαq/11. The Akt/mTOR signaling pathway is typically regulated by the activation of PI3K, which can be induced by receptor tyrosine kinase growth factors or GPCR signaling. Inhibition of the Akt /mTOR pathway can clear dysfunctional organelles and misfolded proteins in the cytoplasm, which drive apoptosis by increasing oxidative stress and endoplasmic reticulum stress [25]. The Akt/mTOR signaling pathway has been extensively documented as a key mediator in the stimuli that induce cardiac hypertrophy, driving both adaptive and maladaptive cardiac growth. Several studies have shown that Akt/mTOR signaling is activated by hypertrophic stimuli such as pressure overload, β-adrenergic stimulation, angiotensin II, and insulin-like growth factor 1 (IGF-1). This pathway plays both adaptive and maladaptive roles in the heart [26,27,28]. Akt was activated via phosphorylation of T308, which further activated the downstream GSK-3β/mTOR/p70S6K signaling pathway to promote the synthesis of hypertrophic markers and fibrin, which in turn led to cardiac hypertrophy and dysfunction [29].

In this study, we investigated the effects of the novel Gαq/11 inhibitor GQ262 on anti-cardiac hypertrophy in both in vitro and in vivo models. We found that GQ262 exerts its anti-hypertrophic and anti-apoptotic effects in cardiomyocytes (CMs) by inhibiting the Akt/mTOR signaling pathway, thereby reducing cardiac fibrosis. This research holds significance for the development of GPCR inhibitors and their potential therapeutic applications in treating cardiac remodeling diseases.

## 2. Results

### 2.1. GQ262 Improves Cardiac Function and Reverses Myocardial Remodeling in TAC Induced HF Mice

**Figure 1:**
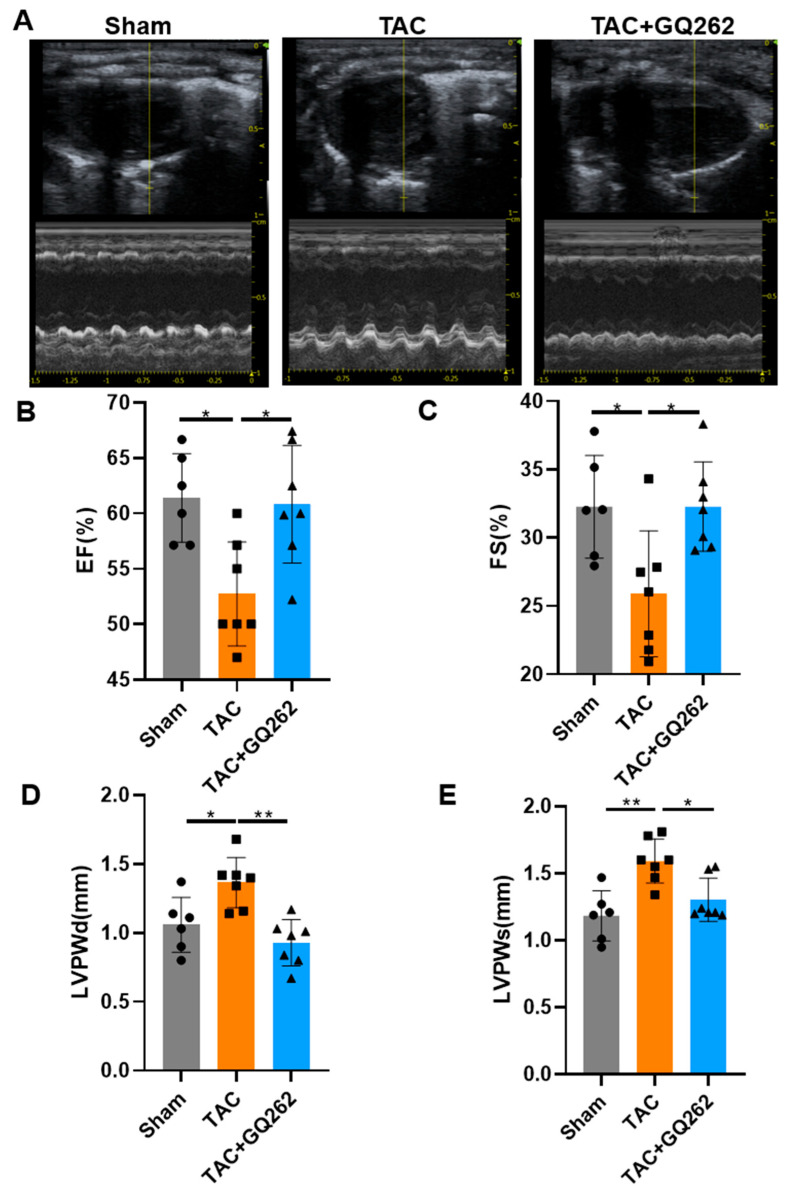
GQ262 improves TAC-induced cardiac dysfunction. (**A**) Representative M-mode echo-cardiography of left ventricular chamber. Bar graphs showing the quantification of (**B**,**C**) LVEF% and LVFS%. (**D**,**E**) Left ventricular posterior wall thickness end-diastole (LVPWd) and left ventricular posterior wall thickness end-systolic (LVPWs). The circular symbols represent the Sham group samples, the square symbols denote the TAC group samples, and the triangular symbols indicate the TAC + GQ262 group samples. Statistical analysis was performed by one-way ANOVA. Data are presented as mean ± SD (n = 6–7). * *p* < 0.05, ** *p* < 0.01.

Left ventricular (LV) functions in all groups after 4 weeks of TAC modeling were evaluated using echocardiography (Figure 1A). Compared to the sham group, mice in the TAC surgical groups exhibited significant LV enlargement and worsening heart function. In contrast, mice treated daily with GQ262 (30 mg/kg) via gavage showed a significant increase in LV EF% and LV FS% (Figure 1B,C). Meanwhile, compared with the saline group, similar improvement trends were observed in other functional indicators, including LV internal diameter at end-diastole and LV internal diameter at end-systole (Figure 1D,E). The results provided evidence that GQ262 treatment effectively reverses the TAC-induced deterioration of cardiac function and structural remodeling in mice.

### 2.2. GQ262 Protects against Cardiac Hypertrophy In Vivo

**Figure 2:**
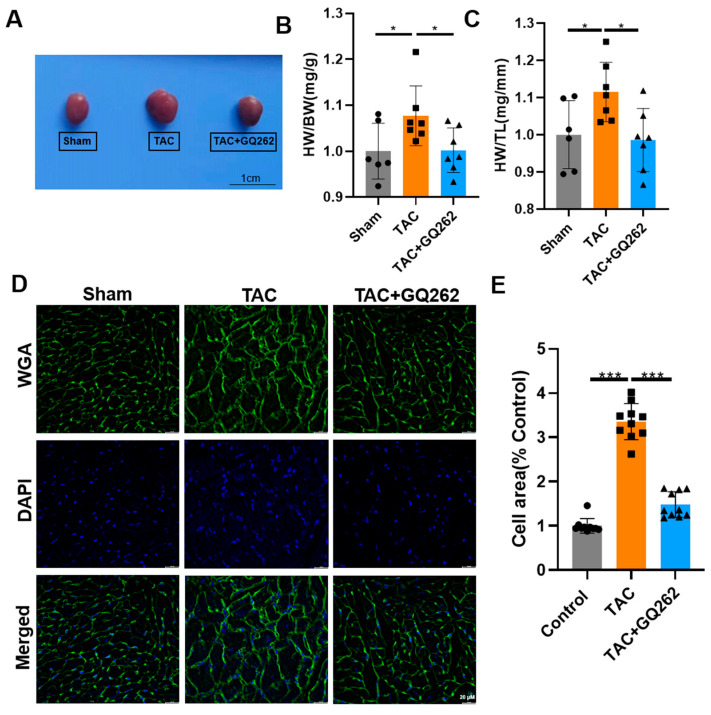
GQ262 inhibits TAC-induced cardiac hypertrophy. (**A**) Image of freshly extracted hearts from mice 4 weeks after sham operation, TAC or TAC + GQ262. Bar graphs showing the quantification of (**B**) Heart weight/body weight ratio (HW/BW). (**C**) Heart weight/tibial length (HW/TL). (**D**) Representative immunofluorescent images of myocardium after staining for WGA (green) and DAPI (blue). (**E**) Quantitative analysis of myocyte area. At least 100 cells measured in different visual fields from 4 samples per group. The quantification of average cross-sectional area of CMs compared with control in the indicated groups are shown in the bar graph. The circular symbols represent the Sham group samples, the square symbols denote the TAC group samples, and the triangular symbols indicate the TAC + GQ262 group samples. Statistical analysis was performed by one-way ANOVA. Data are presented as mean ± SD (n = 6–10). * *p* < 0.05, *** *p* < 0.001.

**Figure 3:**
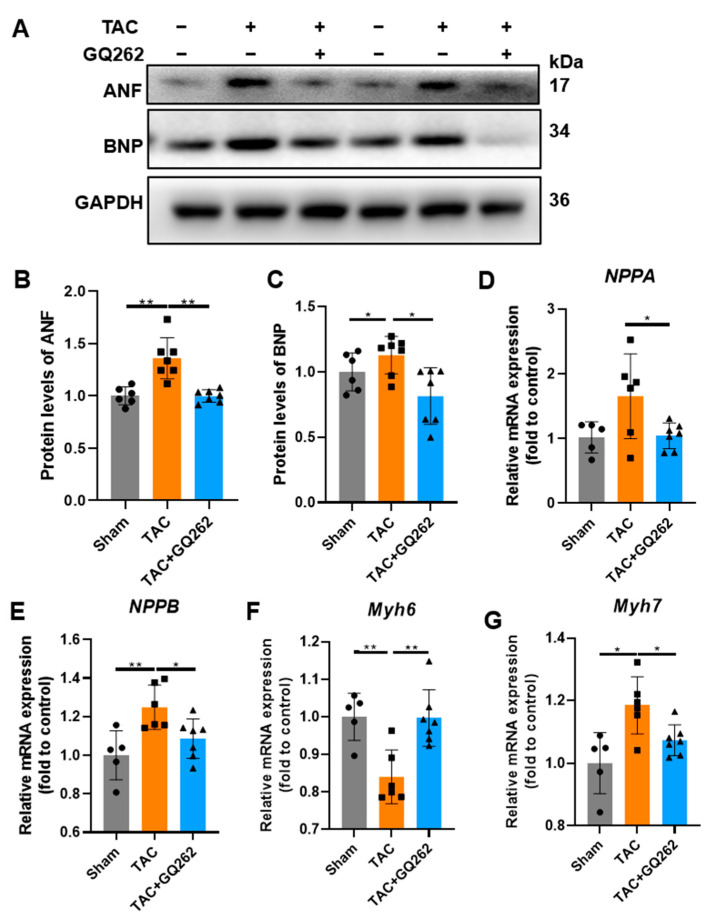
GQ262 inhibits the mRNA and protein levels of hypertrophy-related genes in the TAC-induced mouse heart tissues. (**A**–**C**) Western blot and quantification of BNP and ANF expression levels in heart tissues. (**D**–**G**) Quantification of mRNA levels about ANF, BNP, Myh6 and Myh7. The circular symbols represent the Sham group samples, the square symbols denote the TAC group samples, and the triangular symbols indicate the TAC + GQ262 group samples. Statistical analysis was performed by one-way ANOVA. Data are presented as mean ± SD (n = 5−7). * *p* < 0.05, ** *p* < 0.01.

GQ262 significantly reduced the enlarged heart size and the heart weight to body weight (HW/BW) or the length of the tibial ratio (HW/TL) (Figure 2A–C). WGA staining revealed that GQ262 significantly reduced TAC-induced hypertrophy by decreasing cell volume size (Figure 2D,E). Furthermore, we compared the transcriptional and protein levels of the key markers related to cardiac hypertrophy (ANF and BNP). The TAC-induced increase in ANF and BNP were also reversed by GQ262 (Figure 3A–C). Additionally, these molecular markers were significantly augmented in the TAC group but were normalized in TAC + GQ262 mice (Figure 3D–G). Taken together, these data suggest that hypertrophic CMs could be recovered to a respectively normal size by GQ262 treatment, which may be responsible for the effect of GQ262 in reversing TAC induced hypertrophy.

### 2.3. GQ262 Protects against TAC-Induced Cardiac Fibrosis

**Figure 4:**
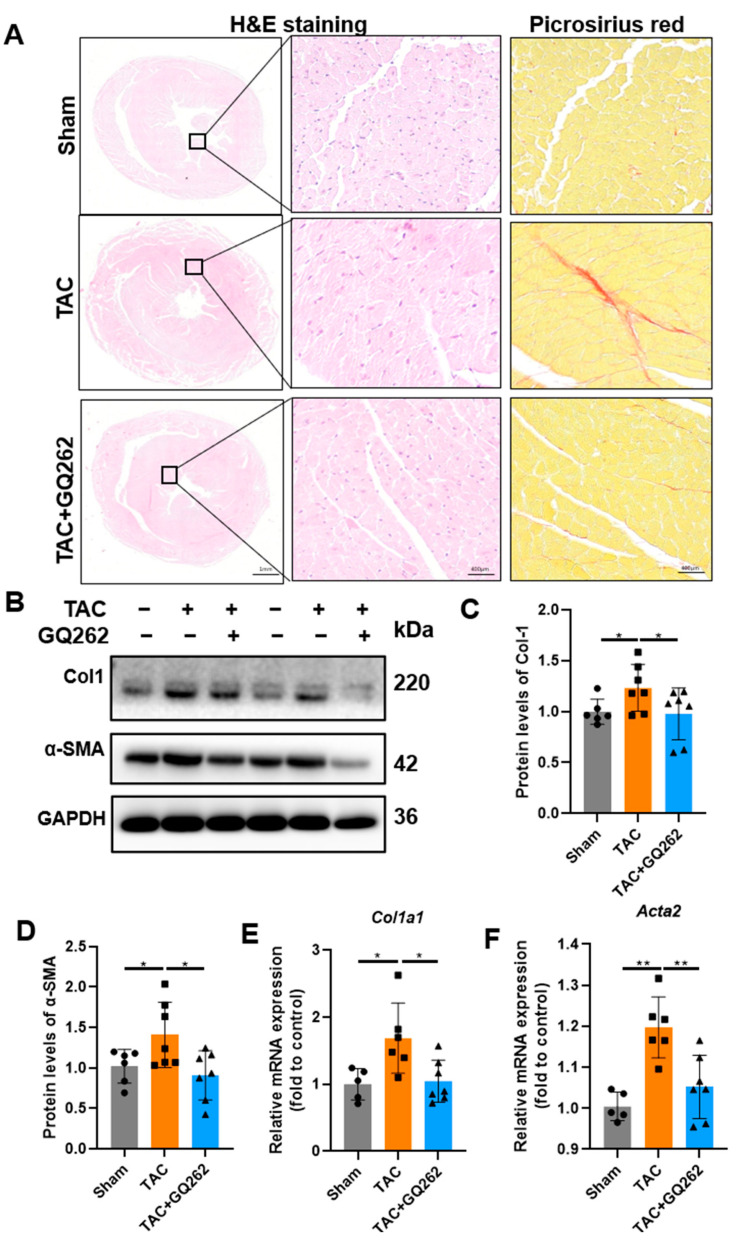
GQ262 inhibits TAC-induced fibrosis. (**A**) H&E and picrosirius red staining of the heart tissues showing the effect of GQ262 on TAC-induced mouse cardiac fibrosis. (**B**–**D**) Western blot and quantification of Col1 and α-SMA levels in the mouse heart tissues. (**E**,**F**) Quantification of mRNA levels about Col1 and α-SMA. The circular symbols represent the Sham group samples, the square symbols denote the TAC group samples, and the triangular symbols indicate the TAC + GQ262 group samples. Statistical analysis was performed by one-way ANOVA. Data are presented as mean ± SD (n = 5–7). * *p* < 0.05, ** *p* < 0.01.

For the assessment of cardiac fibrosis, microscopic images from H&E and Masson’s trichrome staining showed focal myocardial cell necrosis beneath the epicardium, with nuclear condensation or fragmentation and myocardial cell hypertrophy after TAC modeling. Fibroblasts and collagen fibers were arranged in an interlaced pattern. GQ262 significantly reduced the degree of fibrosis (Figure 4A). Nonetheless, we examined the expression levels of fibrosis markers, Col1 and α-SMA. The protein expression levels of these increased in the TAC group. However, treatment with GQ262 significantly reversed the cardiac fibrosis (Figure 4B–D). Furthermore, qPCR analysis indicated that GQ262 treatment blunted TAC-induced upregulation of Col1a1 and Acta2’s mRNA levels (Figure 4E,F). These findings suggest that GQ262 may protect against TAC-induced fibrosis.

### 2.4. GQ262 Inhibits PE-Induced Cardiac Hypertrophy in H9C2 Cells

**Figure 5:**
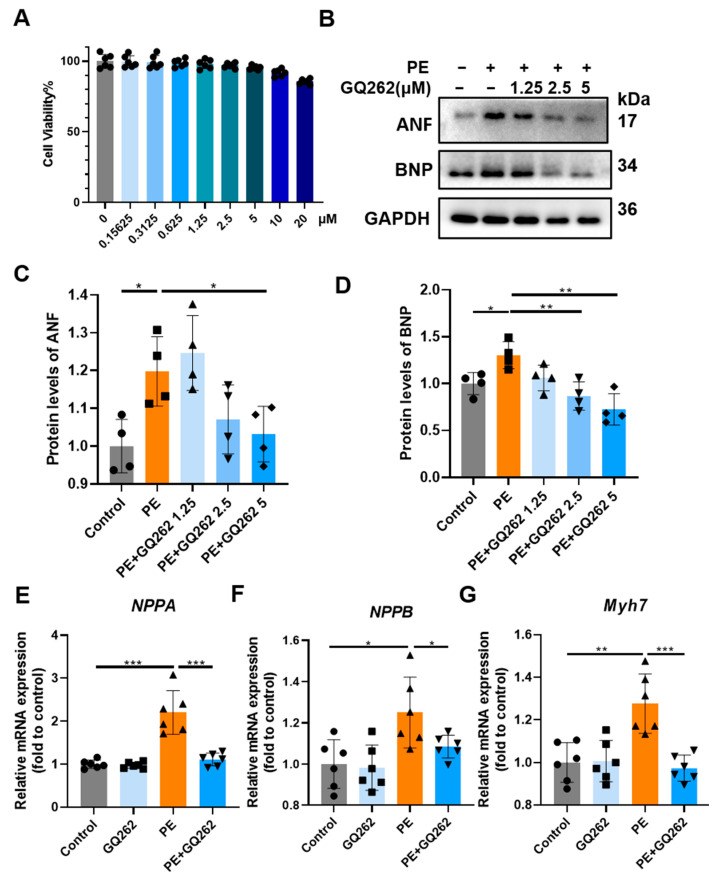
GQ262 inhibits PE-induced cardiomyocyte hypertrophy. (**A**) Cell viability of H9C2 cells after treatment with different concentrations of GQ262. The circular symbols represent the cell viability after treatment with different concentrations of GQ262. (**B**–**D**) Western blot and quantification of ANF and BNP protein levels. The circular symbols represent the Control group samples, the square symbols indicate the PE group samples, and the upright triangle, inverted triangle, and diamond symbols correspond to the different doses of the PE + GQ262 group samples, respectively. (**E**–**G**) Quantification of mRNA levels of ANF, BNP and Myh7. The circular symbols indicate the Control group samples, the square symbols represent the GQ262 group samples, the upright triangles denote the PE group samples, and the inverted triangles represent the PE + GQ262 group samples. Statistical analysis was performed by one-way ANOVA. Data are presented as mean ± SD (n = 5–6). * *p* < 0.05, ** *p* < 0.01, *** *p* < 0.001.

Myocardial H9c2 cells were treated with 0.16–20.00 μmol/L PE for 24 h. When the GQ262 concentration was under 20 μmol/L, there was no significant difference in cell viability compared with the control group (Figure 5A). In the subsequent experiments, doses of 1.25, 2.5 and 5 μmol/L were utilized. Cardiomyocyte hypertrophy is characterized by upregulation of embryonic proteins and genes. Compared with the PE group, treating with 1.25, 2.50 or 5 μmol/L GQ262 decreased the PE-induced protein expression of ANF, BNP in H9C2 cells in a concentration-dependent manner (Figure 5B–D). The mRNA levels of these genes were markedly elevated in the PE group. However, treatment with a high dose of GQ262 significantly decreased the protein and mRNA expression of ANF, BNP and Myh7. (Figure 5E–G). These results suggested that GQ262 alleviates the pathological changes of PE-induced cardiac hypertrophy.

### 2.5. GQ262 Protects against PE and TAC-Induced Apoptosis

**Figure 6:**
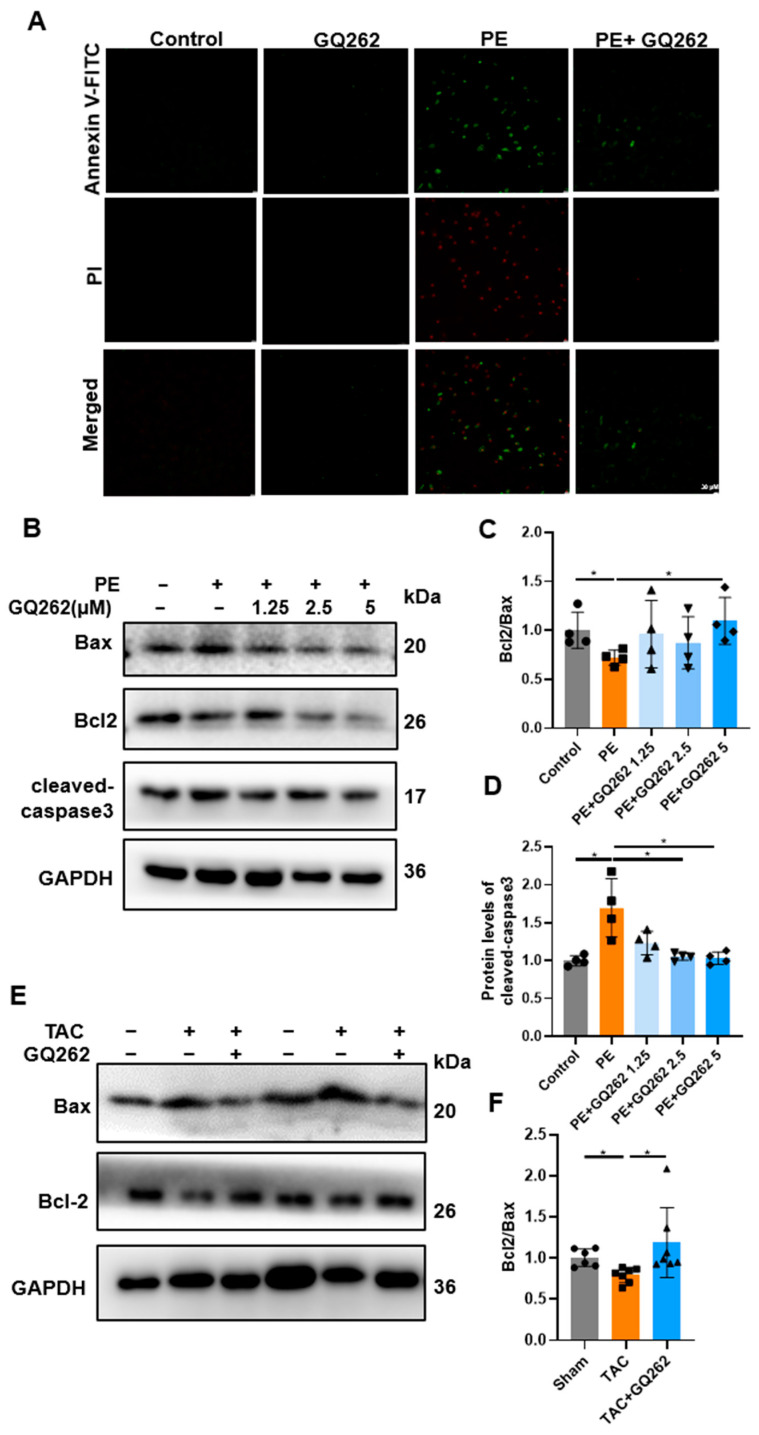
GQ262 inhibits both PE and TAC-induced cardiomyocyte apoptosis. (**A**) Apoptosis was analyzed with Annexin V-FITC/PI staining, and observed under a fluorescence microscope. (**B**–**F**) Western blot and quantification of protein levels of Bcl2/Bax and cleaved caspase-3. The circular symbols represent the control or sham group samples, the square symbols indicate the model group samples, and the upright triangle, inverted triangle, and diamond symbols correspond to the samples treated with different drug doses, respectively. Statistical analysis was performed by one-way ANOVA. Data are presented as mean ± SD (n = 4–7). * *p* < 0.05.

Annexin V-FITC/propidium iodide dual staining was performed on H9C2 cells after treatment with PE and GQ262 for 24 h to observe the inhibitory effect of GQ262 on CMs’ apoptosis. The H9C2 cells were stained and were observed under a fluorescence microscope (Figure 6A). The percentage of apoptotic cells was increased in the PE group. However, after GQ262 treatment, the ratio of apoptotic cells significantly decreased. Next, we analyzed the expression of apoptosis-related proteins in H9C2 cells, including Bcl-2/Bax and cleaved caspase-3. A noticeable decline in Bcl-2/Bax expression caused by PE was observed compared to the control group, with a considerable rise in cleaved caspase-3 levels (Figure 6B–D). The TAC model also upregulates the expression of Bax protein and inhibits the expression of the anti-apoptotic protein Bcl-2 (Figure 6E,F). However, GQ262 treatment significantly reversed these aberrant protein expression trends. Consequently, treatment with GQ262 attenuated PE and TAC-induced cardiomyocyte apoptosis.

### 2.6. GQ262 Attenuates Cardiac Remodeling by Regulating Akt/mTOR Signaling Pathway

**Figure 7:**
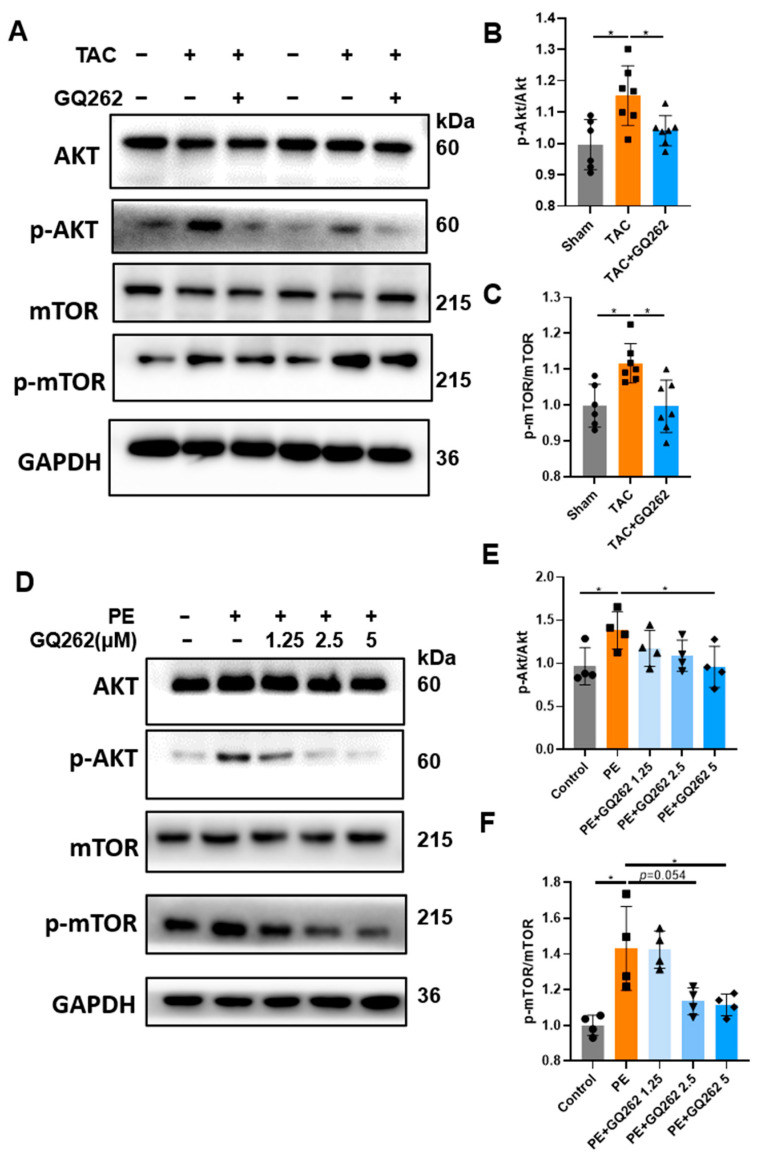
GQ262 decreases the phosphorylation of Akt and its downstream mTOR both in vitro and in vivo. (**A**–**C**) Western blot and quantification of protein levels of Akt/p-Akt in the heart tissues from TAC-induced mice. The circular symbols represent the Sham group samples, the square symbols denote the TAC group samples, and the triangular symbols indicate the TAC + GQ262 group samples. (**D**–**F**) Western blot and quantification of protein levels of mTOR/p-mTOR in the PE-induced H9C2 cells. The circular symbols represent the Control group samples, the square symbols indicate the PE group samples, and the upright triangle, inverted triangle, and diamond symbols correspond to the different doses of the PE + GQ262 group samples, respectively. Statistical analysis was performed by one-way ANOVA. Data are presented as mean ± SD (n = 4). * *p* < 0.05.

The Akt/mTOR signaling pathway is known to be activated during cardiac remodeling. We investigated whether GQ262 inhibits cardiac hypertrophy by suppressing the Akt/mTOR signaling pathway. The inhibitory effect of GQ262 on the phosphorylation of Akt and its downstream target mTOR was confirmed in vivo via Western blot analysis (Figure 7A–C). Subsequently, in H9C2 cells, GQ262 was found to inhibit the phosphorylation of Akt and mTOR in a dose-dependent manner, with maximal efficacy observed at a GQ262 concentration of 5 µmol/L (Figure 7D–F). These results suggest that GQ262 exerts regulatory effects on the Akt/mTOR signaling pathway both in vivo and in vitro.

### 2.7. The Akt Inhibitor Attenuates the Cardioprotective Efficacy of GQ262 against Cardiac Hypertrophy

**Figure 8:**
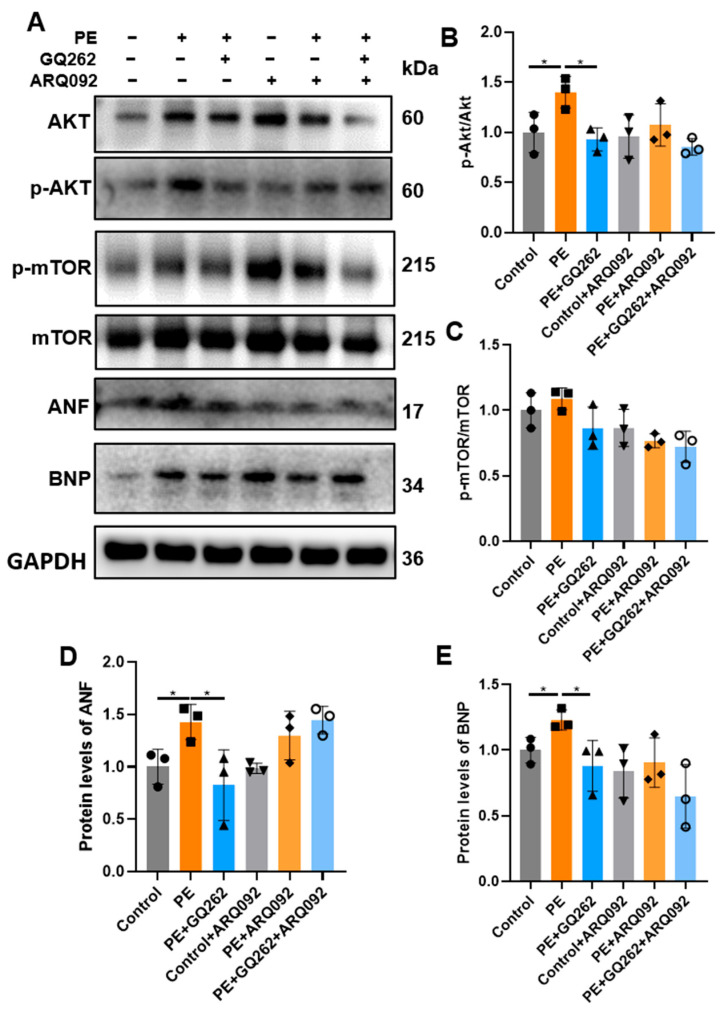
Akt inhibitor abolishes the effect of GQ262 on the PE-induced H9C2. (**A**–**C**) Western blot and quantification of protein levels of Akt/p-Akt and mTOR/p-mTOR in the H9C2 cells with or without ARQ092 treatment. (**D**,**E**) Western blot and quantification of protein levels of ANF and BNP in the H9C2 cells with or without ARQ092 treatment. The circular symbols represent the Control group samples, the square symbols indicate the PE group samples, and the upright triangles denote the PE + GQ262 group samples. The inverted triangles represent the Control + ARQ092 group samples, the diamonds correspond to the PE + ARQ092 group samples, and the rings indicate the PE + GQ262 + ARQ092 group samples. Statistical analysis was performed by one-way ANOVA. Data are presented as mean ± SD (n = 3). * *p* < 0.05.

Subsequently, we investigated whether GQ262 attenuates myocardial hypertrophy through the suppression of the Akt/mTOR signaling pathway. ARQ092, an Akt inhibitor, was used to block the Akt/mTOR pathway. Moreover, 10 μM ARQ092 was added at the same time as PE and GQ262 for 24 h to inhibit the phosphorylation of Akt. The addition of ARQ092 resulted in the inhibition of PE-induced activation of Akt and mTOR, while no further inhibitory effect of GQ262 on the Akt/mTOR signaling pathway was observed (Figure 8A–C). Concurrently, the previously observed inhibitory effects of GQ262 on the hypertrophic markers of ANF and BNP were abrogated (Figure 8D,E). These findings provide further evidence supporting the notion that GQ262 exerts cardioprotective effects against cardiac remodeling by inhibiting the Akt/mTOR pathway.

## 3. Discussion

Heart failure, a leading cause of global mortality, is often triggered by pathological cardiac hypertrophy. Initially, cardiac hypertrophy serves as a compensatory mechanism to reduce wall stress and improve cardiac output [30,31]. However, prolonged hypertrophy eventually leads to a decline in cardiac contractility, progressing to cardiac decompensation and ultimately heart failure [32]. Hypertrophic cardiomyocytes are characterized by an increase in cell size rather than proliferation, as post-mitotic adult cardiomyocytes lack the ability to divide [33]. The heart, consisting of various cell types including cardiomyocytes (which make up 30% of the total cell count but 70 to 80% of the heart’s mass), fibroblasts and others, undergoes hypertrophy primarily through the enlargement of cardiomyocytes [34,35]. Cardiac hypertrophy involves extensive intracellular changes, including pathological metabolism, myocardial fibrosis, oxidative stress, cell death (such as necrosis, apoptosis, and autophagy), inflammation and gene alterations. It also includes extracellular matrix (ECM) changes, such as fibrosis and angiogenesis [36]. In this study, we utilized a TAC-induced mouse model and a PE-induced H9C2 cell model, both of which successfully demonstrated significant hypertrophy. GQ262 significantly inhibited myocardial hypertrophy in both models.

GPCRs are activated by various factors that increase during cardiac stress and HF, with Gαq, a G protein subunit, being crucial in the development of pathological cardiac hypertrophy. Receptors such as AT1R, endothelin receptors and α1-adrenergic receptors are stimulated by hormones like angiotensin II (Ang II). This activation triggers downstream signaling pathways involving proteins such as phospholipase C and MAPKs, which contribute to the progression of cardiac hypertrophy [12]. Studies have demonstrated that manipulating GPCR signaling can affect heart growth. Overexpression of Gαq can induce heart failure, while reduced GPCR signaling is associated with diminished hypertrophy [11]. However, previous researchers have only performed knockdown or overexpression studies, and no specific Gαq/11 inhibitors have been used for the treatment of cardiac hypertrophy because of a lack of safe and effective inhibitors. Additionally, compared to other G proteins, research on Gαq/11 in cardiovascular diseases is insufficient. The Fujisawa Pharma Inc. extracted a natural Gαq/11 inhibitor, FR900359, from *Ardisia crenata*, which was found to have vasodilatory activity on rat aortas [18]. This effect is due to the increased release of NO from endothelial cells at low concentration. Another natural Gαq/11 inhibitor, YM-254890, derived from *Chromobacterium* sp., exhibits vasorelaxant and anti-platelet effects. Unfortunately, YM has been withdrawn by Astellas Pharma Inc. and is no longer available to researchers. According to the literature, YM-254890 inhibits Gαq/11 with an efficiency of 50% at 15 μmol/L [37], while GQ262 achieves a 57.2% inhibition at 10 μmol/L. Neither of these natural inhibitors have been extensively developed due to their limited sources and complex extraction processes [19]. Our collaborator Prof. Xiong’s team previously synthesized the inhibitor GQ127, but its specificity for Gαq/11 inhibition also needs improvement and the compound has a relatively short half-life [20]. GQ262, is a novel Gαq inhibitor that demonstrates excellent safety, enhanced inhibitory activity against Gαq/11, favorable drug-like properties and high oral bioavailability [38], making it a promising candidate for clinical development. We further investigated the potential and mechanisms of GQ262 in the treatment of cardiac hypertrophy and found that GQ262 administration can reverse TAC-induced hypertrophy and its associated decline in cardiac function.

Fibrosis in the heart, marked by the excessive accumulation of ECM proteins such as collagens, fibronectin, matrix metalloproteinases (MMPs) and their inhibitors (TIMPs), is a key feature of various pathological cardiac conditions [39]. Typically, cardiac fibroblasts within the ECM produce and regulate components like collagen types I and III, maintaining a balance between collagen synthesis and degradation [40]. This balance is essential for providing structural support to cardiomyocytes and supporting their mechanical, chemical, and electrical functions [41]. After myocardial injury, the activation of fibroblasts and the subsequent exacerbation of fibrosis occur through several mechanisms. Firstly, myocardium injury leads to the release of pro-inflammatory cytokines, growth factors, and other signaling molecules such as transforming growth factor-beta (TGF-β), platelet-derived growth factor (PDGF), and Ang II [42]. These factors stimulate resident fibroblasts and recruit circulating fibroblast precursors to the site of injury. Activated fibroblasts then differentiate into myofibroblasts, which are characterized by increased production of ECM components, including collagen [43]. This excessive deposition of extracellular matrix leads to myocardial fibrosis, which can impair cardiac function and contribute to the progression of heart failure. Furthermore, the fibrotic activity of cardiac fibroblasts is controlled by Pitx2 signaling derived from CMs, with its expression increasing during cardiac hypertrophy. Pitx2 is considered a downstream transcription factor in the TGF-β signaling pathway in non-muscle cells [44]. Chronic activation leads to abnormal collagen deposition and accumulation, causing mechanical stiffening of the heart. This stiffening impairs both diastolic and systolic function and increases the risk of arrhythmias by disrupting electrical conduction within the heart [45]. GQ262 inhibits hypertrophy, reduces myocardial injury, and decreases the release of fibroblast-activating factors and the excessive deposition of ECM, thereby mitigating the fibrosis process.

It has been suggested that the apoptosis levels of CMs are influenced by the ratio of Bcl-2 to Bax. High expression of Bcl-2 results in an increased Bcl-2/Bax ratio, which inhibits cardiomyocyte apoptosis. Conversely, high expression of Bax leads to a decreased Bcl-2/Bax ratio, promoting cardiomyocyte apoptosis. Caspase-3, a key executor of apoptosis, shows a positive correlation with apoptosis levels. Additionally, caspase-3 can inhibit Bcl-2 expression, while Bcl-2 can reduce caspase-3 levels [46]. Within 24–48 h after cardiac hypertrophy, myocardial apoptosis occurs, exacerbating myocardial damage. Literature indicates that activated Gαq can induce apoptosis in hamster ovary cells and Cos-7 cells, providing a plausible mechanism for the decompensation of hypertrophied hearts in vivo [47]. This study demonstrated that myocardial injury primarily increased Bax levels and decreased Bcl-2 levels, resulting in a marked reduction in the Bcl-2/Bax ratio. GQ262, however, not only increased Bcl-2 levels but also decreased Bax levels, leading to a significantly higher Bcl-2/Bax ratio compared to the model group. GQ262 inhibited cardiomyocyte apoptosis, thereby slowing down the pathological progression of cardiac remodeling.

Existing studies have shown that cardiac hypertrophy can be inhibited by suppressing the Akt/mTOR signaling pathway. For example, resveratrol suppressed chronic intermittent hypoxia-induced cardiac hypertrophy by targeting the Akt/mTOR pathway. Meanwhile, inhibiting the Akt/mTOR pathway improves cardiac function after ischemic damage [48]. The Akt/mTOR pathway is also one of the main signaling pathways downstream of Gαq. Our results indicate that during the anti-cardiac hypertrophy effects of GQ262, the protein levels of p-Akt and p-mTOR are significantly reduced. When the activation of the Akt signaling pathway is inhibited by its inhibitor ARQ092, the anti-cardiac remodeling effects of GQ262 are abolished. These findings suggest that GQ262 exerts its anti-myocardial hypertrophy effects through the inhibition of the Akt/mTOR signaling pathway.

In summary, our study found that GQ262 can inhibit pathological hypertrophy and cardiomyocyte apoptosis, thereby reducing fibrosis. These findings indicate that GQ262 can inhibit cardiac remodeling. Mechanistically, the cardioprotective effects of GQ262 are mediated through the inhibition of AKT/mTOR pathway activation. These findings provide scientific evidence supporting the cardiovascular protective activity of GQ262 and suggest that GQ262 could be a novel therapeutic agent for the treatment of cardiac remodeling.

## 4. Materials and Methods

### 4.1. Chemicals and Reagents

Tribromoethanol (T48402-5G) and tert-butanol (152463) were obtained from Sigma Aldrich (St. Louis, MO, USA), a 27-gauge needle (300841) was obtained from Roche (Basel, Switzerland), wheat germ agglutinin was obtained from Invitrogen (Carlsbad, CA, USA), RIPA buffer was obtained from BOSTER Biological Technology (Wuhan, China), TRIzol reagent (ET111-01) was obtained from Transgen (Beijing, China), a cDNA Synthesis Kit (K1622) was obtained from ThermoFisher (Billerica, MA, USA), and MTT, Annexin V (C1052) and propidium iodide (PI) were obtained from Beyotime (Shanghai, China).

For the western blot analysis, the primary antibody for ANF (ab225844), Col-1(ab34710) and Akt (ab8805) were purchased from Abcam (Cambridge, UK), BNP (sc-271185) was purchased from Santa Cruz Biotechnology (Santa Cruz, CA, USA), p-mTOR (67778-1-Ig), mTOR (66888-1-Ig), GAPDH (60004-1-Ig) and α-SMA (14395-1-AP) were purchased from Proteintech (Chicago, IL, USA), and p-Akt (4060T), Bax (#2772), Bcl2 (3498S) and cleaved caspase3 (9664S) were purchased from Cell Signaling Technology (Danvers, MA, USA).

### 4.2. Cell Culture and Treatment

Mouse NIH/Swiss embryo fibroblast cells (NIH-3T3) or rat embryonic H9C2 cardiomyocytes (H9C2) were obtained from ATCC and maintained in a DMEM medium containing 10% fetal bovine serum (FBS), penicillin (100 μg/mL) and streptomycin (100 μg/mL) at 37 °C with 5% CO_2_. To establish an in vitro hypertrophy cell model, H9C2 cells were starved in the 2% FBS medium for 12 h and then subjected to 200 μM PE for 24 h, and the cells were also simultaneously treated with different doses of GQ262 (0, 1.25, 2.5, 5 µM).

### 4.3. Animal Model of Cardiac Remodeling

Mice (10 wk old, 23–25 g weight) were allowed to adapt to the environment for 1 wk and then randomly divided into three groups (6–7 mice in each group): control, TAC (transverse aortic constriction), and TAC + GQ262. To induce cardiac remodeling, the mice were anesthetized by intraperitoneal injection of 15–17 mg/kg Alfred working fluid (prepared by adding physiological saline to 1.25 mL storage fluid to make a volume of 50 mL) and ventilated via tracheal intubations connected to a rodent ventilator. The Alfred storage fluid was prepared by adding 5 g tribromoethanol and 10 mL tert-butanol. TAC surgery was performed to induce cardiac hypertrophy using a 27-gauge needle, while the sham group underwent the same procedure without aorta constriction. Mice in the TAC + GQ262 group were given GQ262 by gavage (30 mg/kg per day).

### 4.4. Echocardiography Analysis

Transthoracic echocardiography was performed via a 30 MHz probe from VINNO6, Vino Technology (Suzhou, China). Indices reflecting left ventricular contractile function, including left ventricular ejection fraction (EF%) and left ventricular fractional shortening (FS%), were calculated.

### 4.5. Histopathological Analysis

The heart tissues were fixed with a 4% paraformaldehyde solution and then embedded in paraffin. Paraffin sections of 5 μm thickness were prepared and subjected to hematoxylin–eosin (H&E) staining, WGA staining, and picrosirius red staining according to the manufacturers’ instructions. H&E staining and WGA staining were employed to determine the morphology and cardiac myocyte area, while picrosirius red staining was utilized to visualize collagen I (in red or yellow) and collagen III (in green) under a microscope.

### 4.6. Western Blot Analysis

Cells or fresh myocardiums were lysed in RIPA buffer (containing 0.1% PMSF). Proteins were separated by sodium dodecyl sulfate–polyacrylamide gel electrophoresis (SDS-PAGE) using 8–10% gradient gels and transferred onto 0.45 μm polyvinylidene fluoride (PVDF) membranes. The expressions of the target protein were normalized to the glyceraldehyde-3-phosphate dehydrogenase (GAPDH) protein.

### 4.7. Real-Time PCR Analysis

To measure the mRNA expression of hypertrophy, fibrosis and apoptosis-associated markers, the total RNA from the mouse LV cardiac tissues or cells was extracted using TRIzol reagent. Subsequently, the mRNA was reverse-transcribed into complementary DNA (cDNA) using the cDNA Synthesis Kit. The Ct values of the target gene amplifications were determined using SYBR Green-1 Master Mix in an Mx3005P qPCR System (Agilent Technologies, Santa Clara, CA, USA). GAPDH was used as the internal control gene to normalize the target genes. The sequences of primers used in the experiments are shown in Table 1.

### 4.8. Methyl Thiazolyl Tetrazolium Assay for Cell Viability

The H9C2 cells were seeded in a 96-well plate at a density of 5 × 10^3^ cells per well, with 6 wells per group. After attachment, the cells were starved in serum-free medium for 12 h and then treated with GQ262 at concentrations of 0.16 μM, 0.31 μM, 0.63 μM, 1.25 μM, 2.50 μM, 5 μM, 10 μM and 20 μM. Following 24 h of treatment, 20 μL of 0.5% MTT solution was added to each well and incubated at 37 °C for 4 h. After discarding the medium, 150 μL of DMSO was added to each well. Absorbance was measured at 570 nm using a microplate reader (Bio-Rad, Hercules, CA, USA).

### 4.9. Annexin V/PI Staining for Apoptosis

The apoptosis analysis of H9C2 cells were seeded into 6-well plates at an initial density of 4 × 10^6^ cells per well and cultivated in different conditions (PE, PE + GQ262 or control) after starvation processing for 12 h. Twenty-four hours later, the cells were harvested and stained with Annexin V and PI for 15 min in the dark. Results were analyzed with CytExpert 2.5 Software (Pasadena, CA, USA).

### 4.10. Statistical Analysis

All data are presented as the mean ± SEM. Statistical analyses were performed using GraphPad Prism 8.3.0 Software (San Diego, CA, USA). Continuous variables were compared by *t*-test. The comparison between different groups was performed by Student’s *t*-test for two groups or one-way analysis of variance (ANOVA) followed by Bonferroni tests for more than two groups.

## Figures and Tables

**Table 1:** Gene-specific primers used in qPCR.

**Gene**	**Forward Sequence (5′–3′)**	**Reverse Sequence (3′–5′)**
*NPPA*	GCTTCCAGGCCATATTGGAG	GGGGGCATGACCTCATCTT
*NPPB*	GAGGTCACTCCTATCCTCTGG	GCCATTTCCTCCGACTTTTCTC
*C* *ol1* *a1*	GGGTCTATGCCACGATTC	GTGTCCCATGTTGGATTTG
*A* *cta2*	CCCAGACATCAGGGAGTAATGG	TCTATCGGATACTTCAGCGTCA
*Myh6*	GCCCAGTACCTCCGAAAGTC	ATCAGGCACGAAGCACTCC
*myh7*	CAACCTGTCCAAGTTCCGCA	CCTAAGGTGCTGTTTCAAAGGC
*G* *apdh*	TGATGACATCAAGAAGGTGGTGAAG	TCCTTGGAGGCCATGTAGGCCAT

## Data Availability

The datasets generated, coded, and analyzed during the current study are available on request.

## Abbreviations

α-SMA, alpha-smooth muscle actin; ANF, atrial natriuretic factor; ANOVA, analysis of variance; Ars, adrenergic receptors; AS, atherosclerosis; BNP, B-type natriuretic peptide; cDNA, complementary DNA; CF, cardiac fibrosis; CH, cardiac hypertrophy; CMs, cardiomyocytes; Col-1, collagen 1; DAG, diacylglycerol; ECM, extracellular matrix; EF, ejection fraction; FBS, fetal bovine serum; FS, fractional shortening; GAPDH, glyceraldehyde-3-phosphate dehydrogenase; GPCR, G protein-coupled receptors; H&E, hematoxylin–eosin; HF, heart failure; HW/BW, heart weight/body weight; HW/TL, heart weight/tibial length; LV, left ventricular; LVPWd, left ventricular posterior wall thickness end-diastole; LVPWs, left ventricular posterior wall thickness end-systolic; MI, myocardial infarction; MMPs, matrix metalloproteinases; Myh6/7, myosin heavy chain 6/7; PDGF, platelet-derived growth factor; PE, phenylephrine; PKC, protein kinase C; PI, propidium iodide; PSR, picrosirius red; PVDF, polyvinylidene fluoride; TAC, transverse aortic constriction; TGF-β, transforming growth factor-beta; TIMPs, tissue inhibitors of metalloproteinases; WT, wild type; WGA, wheat germ agglutinin.
